# Modulation of Gene Expression in Key Survival Pathways During Daily Torpor in the Gray Mouse Lemur, *Microcebus murinus*

**DOI:** 10.1016/j.gpb.2015.03.001

**Published:** 2015-06-17

**Authors:** Kyle K. Biggar, Cheng-Wei Wu, Shannon N. Tessier, Jing Zhang, Fabien Pifferi, Martine Perret, Kenneth B. Storey

**Affiliations:** 1 Institute of Biochemistry & Department of Biology, Carleton University, Ottawa, ON K1S 5B6, Canada; 2 UMR 7179 Centre National de la Recherche Scientifique, Muséum National d’Histoire Naturelle, 91800 Brunoy, France; 3 Biochemistry Department, Schulich School of Medicine and Dentistry, Western University, London, ON N6A 5C1, Canada; 4 Department of Biology, Genetics Institute, University of Florida, Gainesville, FL 32611, USA; 5 Department of Surgery & Center for Engineering in Medicine, Massachusetts General Hospital & Harvard Medical School, Charlestown, MA 02129, USA; 6 Chemistry and Chemical Engineering Department, Royal Military College of Canada, Kingston, ON K7K 7B4, Canada

**Keywords:** Daily torpor, Primate hypometabolism, PPAR gamma coactivator, Ferritin, Chaperone proteins

## Abstract

A variety of mammals employ torpor as an energy-saving strategy in environments of marginal or severe stress either on a daily basis during their inactive period or on a seasonal basis during prolonged multi-day hibernation. Recently, a few Madagascar lemur species have been identified as the only primates that exhibit torpor; one of these is the gray mouse lemur (*Microcebus murinus*). To explore the regulatory mechanisms that underlie daily torpor in a primate, we analyzed the expression of 28 selected genes that represent crucial survival pathways known to be involved in squirrel and bat hibernation. Array-based real-time PCR was used to compare gene expression in control (aroused) versus torpid lemurs in five tissues including the liver, kidney, skeletal muscle, heart, and brown adipose tissue. Significant differences in gene expression during torpor were revealed among genes involved in glycolysis, fatty acid metabolism, antioxidant defense, apoptosis, hypoxia signaling, and protein protection. The results showed upregulation of select genes primarily in liver and brown adipose tissue. For instance, both tissues showed elevated gene expression of peroxisome proliferator activated receptor gamma (*ppargc*), ferritin (*fth1*), and protein chaperones during torpor. Overall, the data show that the expression of only a few genes changed during lemur daily torpor, as compared with the broader expression changes reported for hibernation in ground squirrels. These results provide an indication that the alterations in gene expression required for torpor in lemurs are not as extensive as those needed for winter hibernation in squirrel models. However, identification of crucial genes with altered expression that support lemur torpor provides key targets to be explored and manipulated toward a goal of translational applications of inducible torpor as a treatment option in human biomedicine.

## Introduction

A strong depression of metabolic rate is a hallmark of torpor phenotypes in a wide variety of mammals that employ daily torpor or multi-day hibernation [1]. This survival strategy conserves an animal’s energy expenditure when resources, such as food and water, are scarce or abiotic conditions are highly unfavorable for normal life [1]. A number of genomics-based studies have profiled the transcriptional regulation of winter hibernation in rodents and black bears, identifying many genes that are differentially expressed during torpor in hibernators [2–4]. Overall findings from these studies show coordinated regulation of cellular processes that are involved in metabolic functions and cytoprotection that are crucial to maintaining the torpor phenotype. To date, notable changes in transcription have been linked with genes involved in energy and fuel utilization processes, such as cell growth, glycolysis, lipid metabolism, molecular transport, and cellular stress response (*e.g.*, unfolded protein response, apoptosis, cell cycle arrest, and antioxidant defense) [2,3,5–7].

For many years, the repertoire of animal hibernation models has been primarily rodents with some studies of bats, bears and marsupials [1–7]. However, recently, it has been discovered that a few small lemur species from Madagascar undergo metabolic rate depression and descend into daily torpor or even multi-day hibernation to support survival during the dry season when food is scarce [8,9]. To our best knowledge, the gray mouse lemur (*Microcebus murinus*) and a few other small Malagasy lemurs represent the only primates with this ability. Indeed, hibernation in lemurs is also novel among mammals, which are the only cases of natural hibernation not accompanied by profound reductions in body temperature [8,9]. Hence, lemurs are the closest species to humans that can undergo naturally-induced hypometabolism. To gain insight into the mechanisms of primate torpor, the present study investigated transcriptional regulation in the gray mouse lemur, by examining the expression of a selected group of genes that have been shown to be regulated in rodent hibernation. Previous studies have shown that although the overall rate of gene transcription is reduced during torpor, expression of some selected genes is upregulated [10]. Examples of these genes include those involved in the regulation of lipid metabolism (fatty acid-binding proteins, peroxisome proliferator-activated receptor gamma coactivator), inhibition of carbohydrate catabolism (pyruvate dehydrogenase kinase), as well as those involved in antioxidant defense (Cu/Zn superoxide dismutase, aflatoxin aldehyde reductase, and heme oxygenase-1) [5,10].

Using real-time PCR, the present study analyzed the expression of 28 genes in five tissues of *M. murinus*. These genes were chosen based on their biological roles and their historical significance in previous hibernation studies (Table 1) [2,3,5–7], which are involved in the regulation of metabolism, apoptosis, hypoxia resistance, protein chaperones and antioxidant defense. By comparing with previous findings from hibernating rodents, understanding the regulatory pattern of these genes during torpor can help to determine if the molecular signatures seen in primate torpor are similar to those observed among non-primate mammals. Our study is the first to provide molecular evidence of transcriptional regulation in primate torpor.

## Results

It has been previously demonstrated that mammalian hibernation typically responds with a relatively conserved set of genes that act to adapt the cell to associated stresses. To compare the torpor response of the lemur to that of other mammalian hibernators that are torpid at characteristically-low *T*_b_, we determined the relative expression of genes previously shown to be torpor-responsive in low *T*_b_ hibernators (Table 1), in selected lemur tissues using custom RT-PCR arrays designed for primate species. The relative mRNA expression levels of 28 genes were measured in five tissues including the liver, kidney, skeletal muscle, heart, and brown adipose tissue (BAT), by comparing control (aroused) and torpid lemurs to determine their transcriptional response during primate torpor. The genes chosen for this study were categorized according to their known cellular roles: metabolism (*i.e.*, fatty acid and glucose metabolism), apoptosis, hypoxia, protein chaperones, and antioxidant defense.

**Figure 1:**
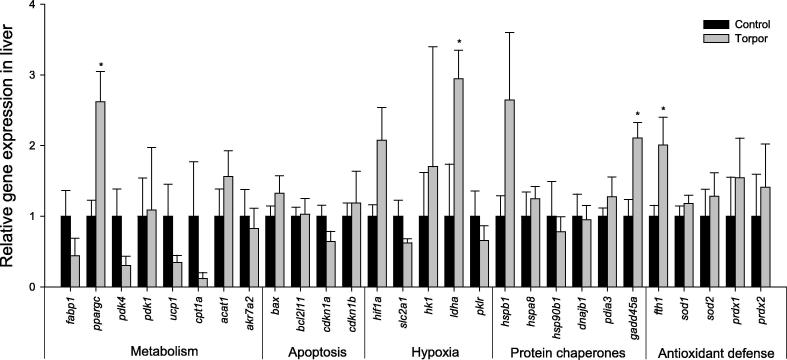
**Relative transcript expression of 28 selected genes in liver of gray mouse lemurs** Histogram shows expression levels of different genes after standardization against *β-actin* expression. Data are means ± SEM, for 3–4 independent samples from separate animals. * Indicates significant difference from the corresponding control (*P* < 0.05). The genes analyzed are listed in Table 1.

**Figure 2:**
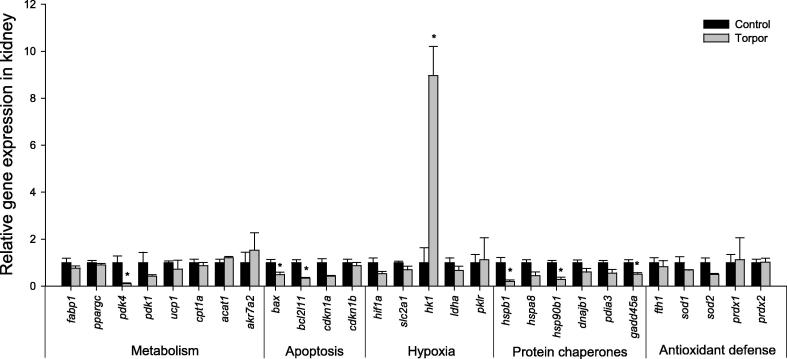
**Relative expression of the 28 genes in kidney of gray mouse lemurs** Expression levels of different genes were normalized against *β-actin* expression. Data are means ± SEM, for 3–4 independent samples from separate animals. * Indicates significant difference from the corresponding control (*P* < 0.05).

In liver tissue, the gene expression of *ppargc* (2.6 ± 0.43-fold), *ldha* (2.95 ± 0.41-fold), *gadd45α* (2.11 ± 0.22-fold), and *fth1* (2.01 ± 0.39-fold) was found to increase significantly during torpor, when compared to controls (Figure 1; *P* < 0.05). No significant changes were observed in any of the other genes studied. Notably, peroxisome proliferator-activated receptor gamma, coactivator (PPARGC), the protein encoded by *ppargc*, functions as a key transcription factor that regulates multiple genes within the lipid metabolism. Upregulation of *ppargc* would thus suggest that controlled regulation of lipid metabolism is likely to be important in torpid regulation of the lemur. In kidney tissue, a significant increase in *hk1* expression (8.97 ± 1.24-fold) was observed, along with a significant decrease in the expression of genes involved in apoptosis and heat shock response including *bax* (to 49 ± 9% of controls), *bcl2l1* (33 ± 3% of controls), *gadd45α* (50 ± 6% of controls), *hspb1* (44 ± 16% of controls), *hsp90b1* (29 ± 9% of controls), and *pdk4* (10 ± 4% of controls) (Figure 2; *P* < 0.05). Importantly, *hk1* regulates the rate-limiting step in glycolysis. Its upregulation could implicate a potential change in glycolytic flux in the kidney, suggesting possibly differential preference of metabolic fuels between tissues during torpor.

**Figure 3:**
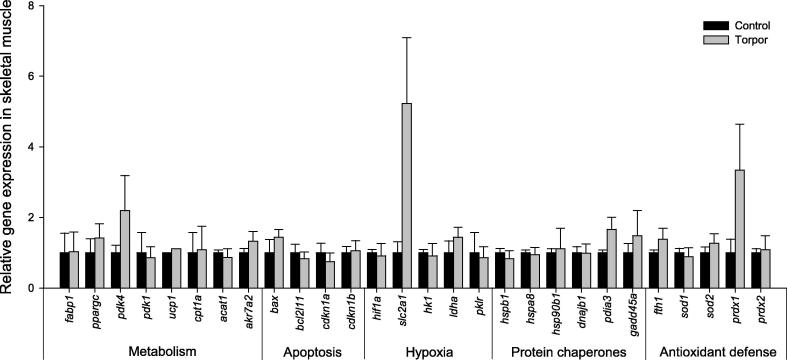
**Relative expression of the 28 genes in skeletal muscle of gray mouse lemurs** Expression levels of different genes were normalized against *β-actin* expression. Data are means ± SEM, for 3–4 independent samples from separate animals.

**Figure 4:**
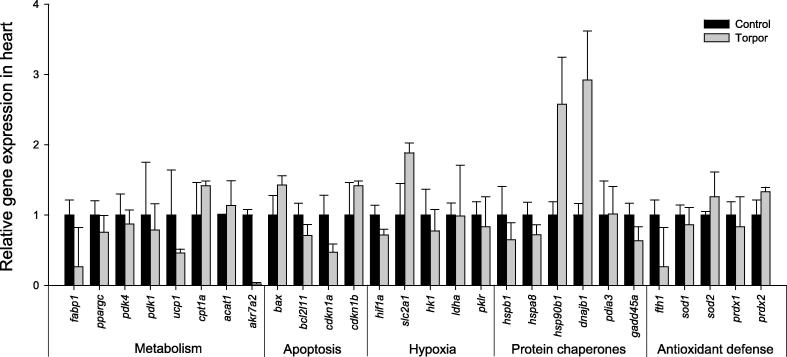
**Relative expression of the 28 genes in the heart of gray mouse lemurs** Expression levels of different genes were normalized against *β-actin* expression. Data are means ± SEM, for 3–4 independent samples from separate animals.

In contrast to liver and kidney tissues, no significant change in gene expression was found in response to torpor in either the skeletal muscle (Figure 3), or heart (Figure 4) tissues. It could be possible that the relative short duration of lemur torpor (compared to other hibernators) might not be sufficient to induce significant changes in expression of genes examined in this study. This would suggest that the torpor-adaptive responses in the muscle tissues are likely unique in lemurs when compared to other hibernators.

**Figure 5:**
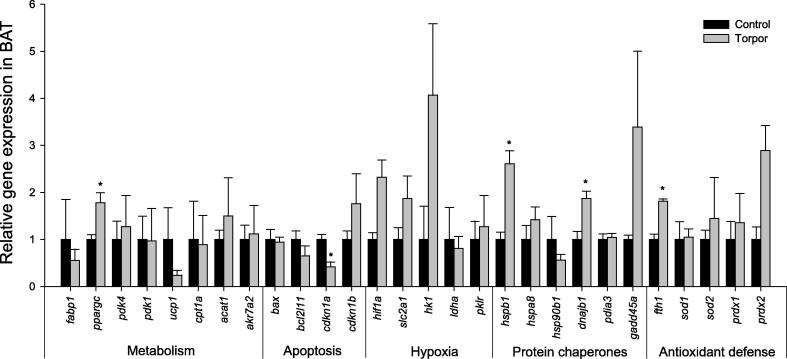
**Relative expression of the 28 genes in BAT of gray mouse lemurs** Expression levels of different genes were normalized against *β-actin* expression. Data are means ± SEM, for 3–4 independent samples from separate animals. * Indicates significant difference from the corresponding control (*P* < 0.05).

In BAT, the expression of *dnajb1* (1.87 ± 0.16-fold), *fth1* (1.82 ± 0.05-fold), *hspb1* (2.61 ± 0.28-fold), and *ppargc* (1.78 ± 0.21-fold) were found to increase significantly in response to torpor, whereas the expression of *cdkn1a* (42 ± 10% of controls) was found to decrease significantly during torpor (Figure 5; *P* < 0.05). Overall, the transcriptional profiling of genes showed tissue-specific patterns in key cellular processes that regulate metabolic functions and stress responses.

## Discussion

This study of gene expression is poised as one of the initial steps in comparing classical models of torpor-hibernation (*e.g.*, squirrels and bats) to the torpor-response of a primate (*i.e.*, the lemur). The genes selected for analysis in the present study were previously shown to be involved in the torpor response of hibernating arctic ground squirrels (*Urocitellus parryii*), a species that can sustain continuous torpor for many days/weeks with core body temperature falling as low as −3 °C [11]. By contrast, the gray mouse lemurs analyzed in the present study were held under conditions where they exhibited daily torpor during their non-active hours (they are nocturnal) and showed relative small decreases in body temperature (nadir *T*_b_ was 30.8 ± 1.6 °C for the 4 animals in the torpor group). Interestingly, we observed unique patterns of tissue-specific gene expression in the torpid lemur, when compared to a classical model of mammalian hibernation. Although only expression of some selected genes was upregulated during lemur torpor, many of the proteins encoded by these genes play important survival and adaptational functions.

A previous study documented the expression of heat shock proteins (HSPs) during torpor in the thirteen-lined ground squirrel (*Ictidomys tridecemlineatus*) [12]. A significant change in the protein expression of HSP70 was detected in the liver, kidney, heart, intestine, or skeletal muscle between active and torpid squirrels. In addition, the abundance of 75 kDa glucose regulatory protein (GRP75) protein was found to be significantly higher in liver, skeletal muscle, and intestine of torpid squirrels when compared to active squirrels [12]. Nonetheless, no significant changes in *hsp70*, also known as (AKA) *hspa8*, mRNA expression were detected between euthermic and torpid states in any of the examined tissues of the gray mouse lemur (Figures 1–5), which is different from the significant HSP70 protein response seen in torpid thirteen-lined ground squirrels. It should be noted that the torpor-responsive differences in expression of this chaperone between the two species may result from a decrease in HSP70 auto-proteolysis during periods of cold torpor in the hibernator, and not from changes in gene expression [13]. Interestingly, expression of two other chaperone-encoding genes, including *dnajb1* (AKA *hsp40*) encoding the partner protein of HSP70 and *hspb1* (AKA *hsp27*), was elevated in response to torpor in BAT, the thermogenic tissue (Figure 5). This may be a reflection of the necessary physiological role that BAT plays in metabolism and energy supply, along with regulating non-shivering thermogenesis during arousal from torpor.

The hypoxia inducible factor (HIF-1) transcription factor is well known to respond to low oxygen levels and has been established to function in the adaptation and protection of cells to hypoxia-related damage [14]. The protection role of HIF-1 includes the upregulation of genes that are necessary to improve oxygen delivery and to enhance the cellular rate of anaerobic glycolysis [15]. A recent study reported differential *hif-1α* gene expression in the little brown bat (*Myotis lucifugus*) and the thirteen-lined ground squirrel in response to hibernation [16]. These opposing patterns of *hif-1α* expression suggest dependence on hibernacula and its oxygen availability (*i.e*., overwintering in caves *vs.* underground burrows), rather than a torpor-specific response [16]. *hif-1α* mRNA levels were increased in the skeletal muscle and liver tissues of little brown bat, as well as in the liver tissue of the squirrel. In contrast with these hibernators, *hif-1α* mRNA levels did not change during torpor in any of the lemur tissues examined. However, expression of *ldha* (encoding lactate dehydrogenase), a downstream gene target of HIF-1α, was upregulated in response to torpor in lemur liver (Figure 1), suggesting that the activity of HIF-1α may be regulated post-transcriptionally, such as that reported for other hibernating animals [16], although it is also possible that *ldha* may be regulated by another transcription factor, such as the cAMP-responsive element-binding protein CREB.

Similar to *ldha* expression, liver was the only tissue to display a torpor-response by *gadd45α*, which encodes GADD45α. GADD45α is an integral component of the stress response and is typically involved in arresting the cell cycle under stressful conditions, which are not favorable for growth or cause DNA damage [17]. Although no comparable studies exist for other models of torpor, a similar response has been seen to oxygen deprivation in anoxia-tolerant red-eared slider turtles (*Trachemys scripta elegans*) [18]. In this regard, it is possible that the increased expression of *gadd45α* is part of the hypometabolic program that is implemented during periods of environmental stress.

The generation of free radicals, such as superoxide (O_2_^−^), can be coupled with changes in metabolic rate. Key enzymatic players in the defense mechanism against reactive oxygen species include peroxiredoxins (antioxidant enzymes that play a major role in the decomposition of H_2_O_2_ to H_2_O and O_2_) and superoxide dismutases (both Mn-SOD, the mitochondrial isoform and CuZn-SOD, the cytosolic isoform) [19,20]. There are also several auxiliary proteins that are involved in the antioxidant defense system, including ferritin (composed of heavy and light chains), which is commonly induced under stress conditions. Ferritin is an iron storage protein that sequesters iron in cells so as to minimize the free Fe^3+^ available to catalyze the Haber–Weiss reaction. A recent study explored the regulation of peroxiredoxins and their contribution to antioxidant defense during torpor in thirteen-lined ground squirrels*.* It was found that the expression of peroxiredoxin (*prdx2*) mRNA increased by 1.7- and 3.7-fold in the BAT and the heart, respectively, during squirrel torpor [21]. Although our study failed to see any torpor-responsive changes in *prdx1* or *prdx2* expression in the lemur, a significant elevation of *fth1* transcripts was identified in both liver and BAT (Figures 1 and 5). The increased *fth1* expression suggests a key role for ferritin in the torpid lemur for iron storage as one mechanism for protection from iron catalyzed oxidative damage.

The switch from carbohydrate to lipid based metabolism is one of the most significant changes typically observed during torpor [11,22]. In many hibernating species, animal do not ingest food for several months, switch to a primary reliance of stored lipids for fuel, and strictly conserve existing carbohydrate reserves for tissues that cannot utilize lipids. Recent microarray analyses of Arctic ground squirrels have shown increased expression of genes involved in fatty acid metabolism during torpor [11]. In contrast, results from our study found changes in genes associated with an increase in glycolytic rate during torpor in the gray mouse lemur. The gene expression of *ppargc*, which encodes peroxisome proliferator-activated receptor gamma coactivator, was elevated significantly in both liver and BAT of torpid lemurs (Figures 1 and 5). *ppargc* is associated with PPAR activation, resulting in lipid uptake in adipocytes and non-oxidative glucose metabolism. The increase in *ppargc* expression in these tissues may function to increase lipid catabolism in BAT, while shutting down pyruvate oxidation in the liver [5]. Additionally, expression of genes such as hexokinase (*hk1*) and *ldha* was upregulated in kidney and liver, respectively (Figures 1 and 2). It is possible that an increase in rate-limited enzyme expression is a preparatory change take place for torpor exit when glycolytic rate can increase. Indeed, glycolytic rate is known to increase quickly during arousal in hibernators such as the deer mouse (*Peromyscus maniculatus*), where the respiratory quotient (RQ) values rise from ∼0.75 during torpor to ∼1.0 upon the transition to arousal [23]. As these are direct measurements of metabolic rate and fuel utilization, it is possible that gene changes take place during torpor to accommodate the glycolytic demand during arousal.

## Conclusion

In summary, the present study provides insight into the regulation of selected genes that are involved the torpor response in species that are studied as models of mammalian hibernation. Overall results for lemurs indicate an increase in the expression of genes that are involved in fatty acid metabolism and antioxidant defense pathways, with limited correlation to the gene expression changes known to accompany hibernation in ground squirrels. These results provide an indication that the molecular mechanisms of torpor may not be as conserved as previously assumed. Future studies utilizing transcriptomic analysis of gene expression in the gray mouse lemur could greatly improve current scientific knowledge in the field and identify key targets for manipulation if research on natural models of hypometabolism is to be translated into applications that could improve human health.

## Materials and methods

### Animals

Gray mouse lemurs used for this study consisted of 8 adult females (2–3 years of age) born in the authorized breeding colony at the National Museum of Natural History (Brunoy, France; European Institution Agreement # D91-114-1). All experiments were carried out as described in greater detail by Biggar and his colleagues [24]. Briefly, control (aroused) animals were sacrificed at the end of a daily torpor bout (*i.e.*, after spontaneous rewarming, *T*_b_ 35–36 °C), whereas torpid mouse lemurs were sacrificed during a torpor bout (when *T*_b_ was at its minimum, 30–33 °C). All animal experiments were performed in accordance with the Principles of Laboratory Animal Care (National Institutes of Health publication 86–23, revised 1985) and the European Communities Council Directive (86/609/EEC). Tissue samples were rapidly excised and immediately frozen in liquid nitrogen. Frozen samples were packed in dry ice and air freighted to Carleton University where tissues were stored continuously at −80 °C until use. All imported tissues were logged as per the Convention on International Trade in Endangered Species of Wild Fauna and Flora (CITES) regulations (export permit No: FR1009118231-E and import permit No: 10cA02291/QWH).

### Total RNA isolation

Total RNA was isolated from the liver, kidney, skeletal muscle, heart, and BAT of euthermic and torpid lemurs with standard procedures using Trizol™ (Invitrogen, Waltham, MA). RNA quality was assessed by the 260/280 nm ratio as well as gel electrophoresis on a 1% agarose gel stained with 1× SYBR Green I (Invitrogen) to check for integrity of the 18S and 28S rRNA bands. All RNA samples were diluted to the concentration at 1 μg/μl using DEPC-treated ddH_2_O.

### Gene expression analysis

A RT^2^ First Strand kit (Cat. No 330401) and a custom-designed RT^2^ Profiler PCR array (Cat. No 330131) were obtained from SABiosciences (Mississauga, ON). Gene info used for primer design is listed in Table S1. For first-strand cDNA synthesis, 1 μg of total RNA was reversely transcribed in a final volume of 10 μl according to manufacturer’s instructions. The cDNA was diluted to 100 μl by adding RNase-free water and stored at −20 °C. PCR was performed using a BioRad MyIQ2 thermocycler (BioRad, Hercules, CA). For one 48-well experiment, 1225 μl of 1 × PCR Master Mix and 49 μl of diluted cDNA were combined in a microcentrifuge tube and an aliquot of 25 μl was added to each well. Profiler PCR arrays were run as indicated by the manufacturer (SABiosciences). Melting curves were acquired for all samples for quality control purposes.

### Statistics

Endogenous control *β*-*actin* (*actb*) was present on each PCR array and used for data standardization. Each cycle threshold (Ct) was normalized to the Ct of the endogenous control. The comparative ΔΔCt method was used to calculate relative quantification of gene expression. Statistical significance of the gene expression difference between the control and torpor samples was assessed using the Student’s *t*-test with SigmaPlot V.11 software. Significant differences were determined at *P* < 0.05.

## Authors’ contributions

All authors contributed to the conception and design of the project and to the editing of the manuscript. MP and FP carried out the animal experiments and KKB, CWW, SNT and JZ conducted biochemical assays. Data analysis and assembly of the draft manuscript was carried out by KBS, KKB and CWW. All authors read and approved the final manuscript.

## Competing interests

The authors have declared no competing interests.

## Figures and Tables

**Table 1:** Genes analyzed in this study and their known roles in mammalian hibernation

Function	Gene symbol	Protein name	Species examined	Refs.
Metabolism	*fabp1*	Fatty acid binding protein 1	Little brown bat (*Myotis lucifugus*), Thirteen-lined ground squirrel (*Ictidomys Tridecemlineatus*), Arctic ground squirrel (*Urocitellus parryii*)	[11,25–28]
	*ppargc*	Peroxisome proliferator activated receptor gamma, coactivator 1 alpha	*M. lucifugus*, *I. tridecemlineatus*	[5,29]
	*pdk4*	Pyruvate dehydrogenase kinase, isozyme 4	*I. tridecemlineatus*	[30]
	*pdk1*	Pyruvate dehydrogenase kinase, isozyme 1	*U. parryii*	[27]
	*ucp1*	Uncoupling protein 1	*U. parryii*	[31]
	*cpt1a*	Carnitine palmitoyltransferase 1A	*U. parryii*	[27]
	*acat1*	Acetyl-CoA acetyltransferase 1	*U. parryii*	[11,27]
	*akr7a2*	Aflatoxin B1 aldehyde reductase member 2	*U. parryii*	[11]

Apoptosis	*bax*	Bcl-2-like protein 4	Golden hamster (*Mesocricetus auratus*)	[32]
	*bcl2l1*	Bcl-2-like protein 1	*I. tridecemlineatus*	[33]
	*cdkn1a*	Cyclin-dependent kinase inhibitor 1A	*I. tridecemlineatus*	[34]
	*cdkn1b*	Cyclin-dependent kinase inhibitor 1B	*I. tridecemlineatus*	[34]

Hypoxia	*hif1a*	Hypoxia-inducible factor 1 alpha	*M. lucifugus*, *I. tridecemlineatus*	[15,16]
	*slc2a1*	Glucose transporter protein type 1	*U. parryii*	[11]
	*hk1*	Hexokinase 1	Richardson’s ground squirrel (*U. richardsonii*)	[35]
	*ldha*	Lactate dehydrogenase A	*U. parryii*, *I. tridecemlineatus*	[11,28]
	*pklr*	Pyruvate kinase, L and R type	*I. tridecemlineatus*	[28]

Protein chaperones	*hspb1*	Heat shock 27 kda protein	*U. parryii*	[11]
	*hspa8*	Heat shock 70 kda protein	*U. parryii*	[11]
	*hsp90b1*	Heat shock 90 kda protein beta, member 1	*U. parryii*, *I. tridecemlineatus*	[11,28]
	*dnajb1*	Heat shock 40 kda protein	*U. parryii*	[11]
	*pdia3*	Glucose regulated protein, 58 kda	*I. tridecemlineatus*	[28]
	*gadd45a*	Growth arrest and DNA-damage-inducible, alpha	*U. parryii*	[11]

Antioxidant defense	*fth1*	Ferritin, heavy polypeptide 1	*U. parryii*	[11]
	*sod1*	Superoxide dismutase 1, soluble	European ground squirrel (*I. citellus*)	[36]
	*sod2*	Superoxide dismutase 2, mitochondrial	*I. citellus*	[36]
	*prdx1*	Peroxiredoxin 1	*U. parryii*, *I. tridecemlineatus*	[11,21]
	*prdx2*	Peroxiredoxin 2	*U. parryii*, *I. tridecemlineatus*	[11,21]

*Note: Urocitellus parryii* and *U. richardsonii* is the synonym of *Spermophilus parryii* and *S. richardsonii*, respectively, whereas *Ictidomys tridecemlineatus* and *I. citellus* is the synonym of *Spermophilus tridecemlineatus* and *S. citellus*, respectively.

## Supplementary material

Table S1 Sequence information for genes amplified in this study
